# *N*-(3,5-Di­chloro-4-hy­droxy­phen­yl)acetamide

**DOI:** 10.1107/S2414314626000751

**Published:** 2026-01-29

**Authors:** Rao M. Uppu, Frank R. Fronczek

**Affiliations:** a Department of Environmental Toxicology Southern University and A&M College,Baton Rouge Louisiana 70813 USA; b Department of Chemistry Louisiana State University,Baton Rouge Louisiana 70803 USA; University of Aberdeen, United Kingdom

**Keywords:** crystal structure, acetamino­phen, chlorinated acetamino­phen

## Abstract

The title compound, C_8_H_7_Cl_2_NO_2_, crystallizes in the triclinic space group *P*1 with three mol­ecules in the asymmetric unit. Two of them are essentially planar, with mean deviations of 13 non-hydrogen atoms of 0.029 and 0.030 Å, and differ only in the conformation of the O—H hydrogen atom. The third mol­ecule is quite nonplanar, with the CCNO acetamide plane forming a dihedral angle of 67.56 (5)° with the remainder of the mol­ecule. In the extended structure, the two almost planar mol­ecules form anti­parallel hydrogen-bonded chains through N—H⋯O inter­actions of the acetamide substituents. The N—H group of the nonplanar mol­ecule donates a bifurcated hydrogen bond to the O—H group and a Cl substituent of one of the planar mol­ecules. The O—H groups of all mol­ecules form inter­molecular hydrogen bonds to other O—H groups or carbonyl O atoms. Together, the hydrogen bonds generate a three-dimensional network.

## Abstract 2

The title compound contains three mol­ecules in the asymmetric unit linked by numerous hydrogen bonds.

## Graphical abstract

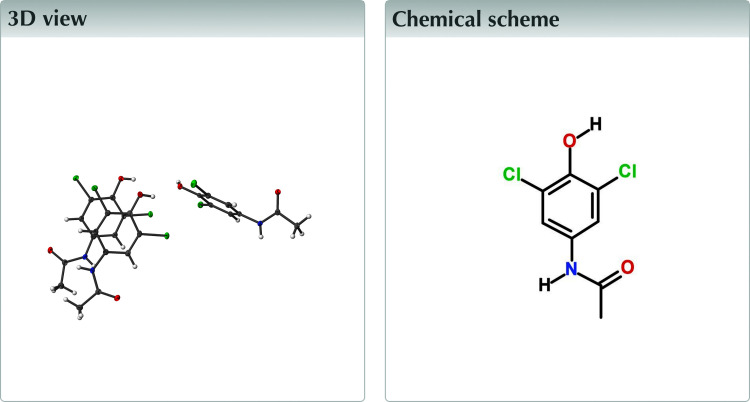

## Structure description

The title compound, C_8_H_7_Cl_2_NO_2_ (**I**), is one of the two well characterized chlorination products formed when acetamino­phen [*N*-(4-hy­droxy­phen­yl)acetamide, C_8_H_9_NO_2_], also known as paracetamol, reacts with hypo­chlorous acid–hypochlorite (HOCl/^−^OCl; p*K*_a_ ≃ 7.5) under mildly oxidative, near-neutral pH conditions (Bedner & MacCrehan, 2006 ▸). Ring-chlorinated products of this type have been detected when wastewater and surface water samples spiked with environmentally relevant concentrations of acetamino­phen were subjected to chlorine-based disinfection (Cao *et al.*, 2016 ▸; Kolpin *et al.*, 2002 ▸; Paíga *et al.*, 2025 ▸). Although the trichlorinated derivative of acetamino­phen does not form under these conditions, the mono- and di­chloro-substituted products are typically accompanied by *p*-benzo­quinone imine, *p*-benzo­quinone, and several high-mol­ecular-weight species with *m*/*z* values between 320 and 610 (Bedner & MacCrehan, 2006 ▸; Glassmeyer & Shoemaker, 2005 ▸; Li *et al.*, 2022 ▸). These transformation products, particularly *p*-benzo­quinone imine and *p*-benzo­quinone, are generally perceived to possess greater toxicol­ogical potency, prompting the adoption of combined and advanced oxidation processes for their efficient removal and detoxification in treated wastewaters (Dahlin & Nelson, 1982 ▸; Postigo & Richardson, 2014 ▸; Qutob *et al.*, 2022 ▸; Phong Vo *et al.*, 2019 ▸).

Similar to those described for HOCl/^−^OCl-mediated oxidations (Bedner & MacCrehan, 2006 ▸), the myeloperoxidase–H_2_O_2_–Cl^−^-acetamino­phen system may generate various chlorinated products, including the title compound (Van Zyl *et al.*, 1989 ▸). While *N*-(3,5-di­chloro-4-hy­droxy­phen­yl)acetamide may serve as a biomarker of chlorination, the compound itself could also pose a significant toxicological concern. Based on linear free-energy relationships and Hammett substituent principles, p*K*_a_ of (**I**) is predicted to be approximately 2.0–2.3 units lower than that of acetamino­phen (pK_a_ ≃ 9.5), since each chlorine substituent in the *ortho* position to the phenolic –OH typically lowers the pK_a_ by about 1.0–1.2 units through strong inductive (–I) effects and stabilization of the phenoxide anion (Hansch *et al.*, 1991 ▸; Perrin *et al.*, 1981 ▸). Accordingly, with an expected pK_a_ in the range of 7.2–7.5, the title compound falls squarely within the classical ‘uncoupler window’ (pK_a_ 4–8; Heytler & Prichard, 1962 ▸). Thus, at physiological pH (7.4), roughly half of the mol­ecules would be deprotonated and half protonated; the likely membrane-permeable properties of (**I**) would therefore favor its behavior as a protonophoric uncoupler, capable of dissipating the mitochondrial protonmotive force that drives ATP synthesis from ADP and inorganic phosphate.

In essence, the stabilization of the phenoxide anion by the two Cl substituents through inductive and intra­molecular hydrogen-bonding effects, together with their likely enhanced lipophilicity and minimal steric hindrance, could render this compound an effective mitochondrial uncoupler. To provide an unambiguous structural basis for these chemical and biological considerations, single crystals of (**I**) were grown from aqueous solution and analyzed by single-crystal X-ray diffraction.

**Figure 1:**
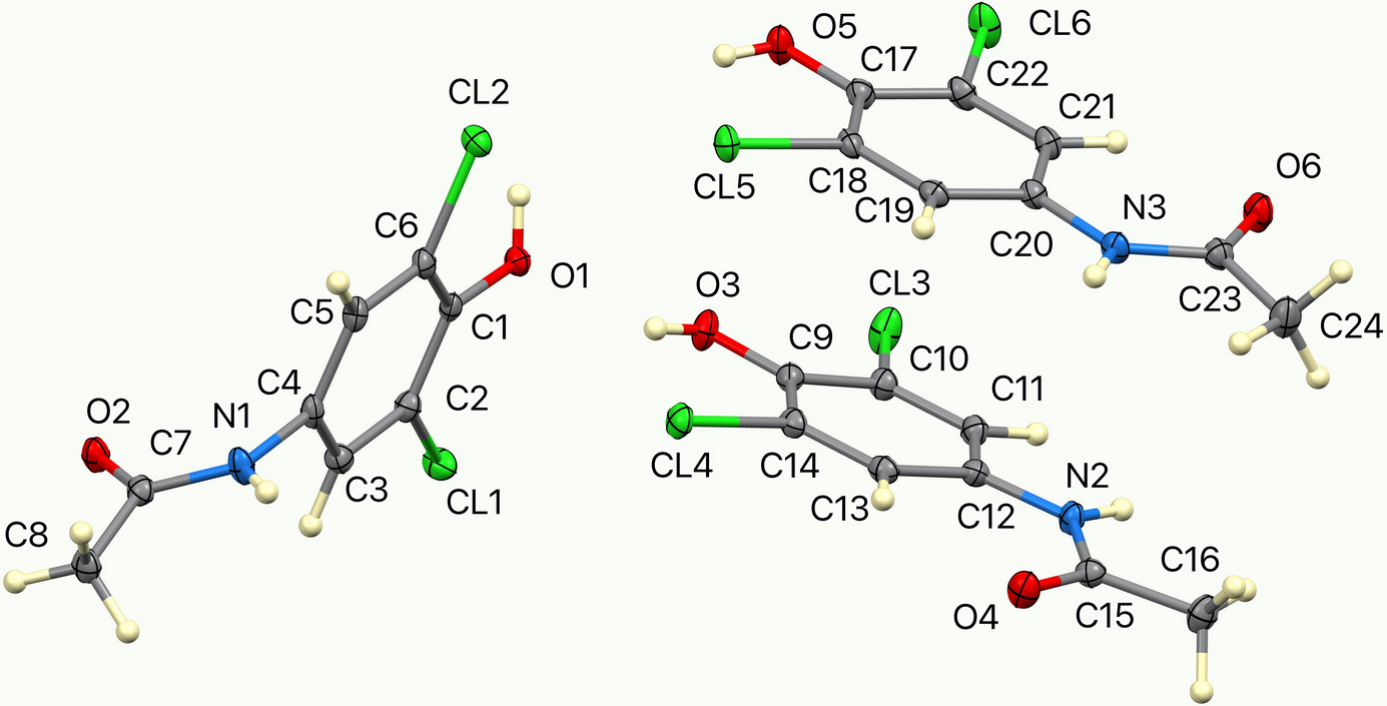
The mol­ecular structure of (**I**), shown with displacement ellipsoids at the 50% probability level.

Compound (**I**) crystallizes in the triclinic space group *P*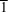 with three independent mol­ecules in the asymmetric unit (Fig. 1 ▸). Two of these mol­ecules, containing atoms N2 and N3, are essentially planar, with mean deviations of their 13 non-hydrogen atoms of 0.029 and 0.030 Å, respectively, and are nearly parallel, forming a dihedral angle of 7.51 (4)°. They differ only in the conformation of the OH hydrogen atom (C14—C9—O3—H30H = 3.09°; C18—C17—O5—H50H = −6.9°). The third mol­ecule containing atom N1 is distinctly nonplanar, with the C7/C8/N1/O2 acetamide plane forming a dihedral angle of 67.56 (5)° with the remainder of the mol­ecule. In the three mol­ecules, the C—Cl distances range from 1.7197 (17) to 1.7381 (18) Å (mean 1.7311 Å), and the C—OH distances range from 1.351 (2) to 1.359 (2) Å (mean 1.354 Å).

**Figure 2:**
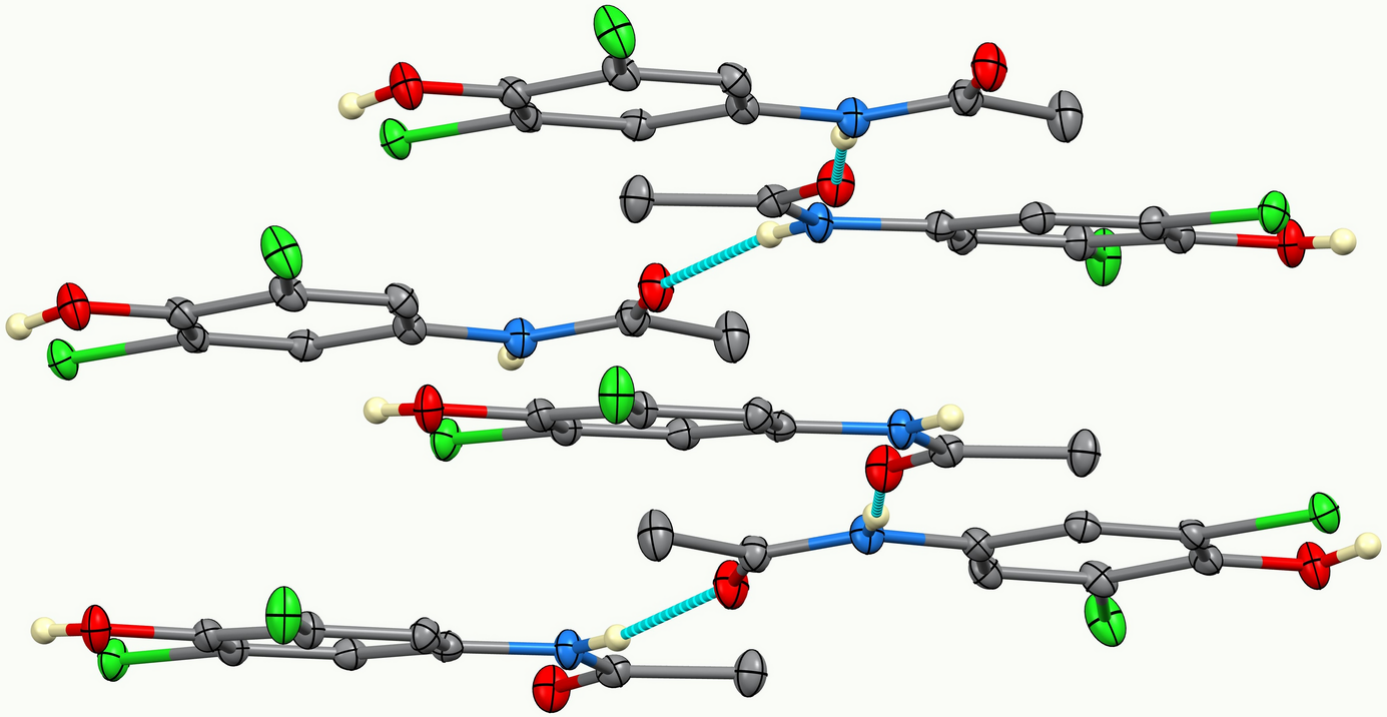
Inter­molecular hydrogen-bonding network in the crystal structure of (**I**).

**Figure 3:**
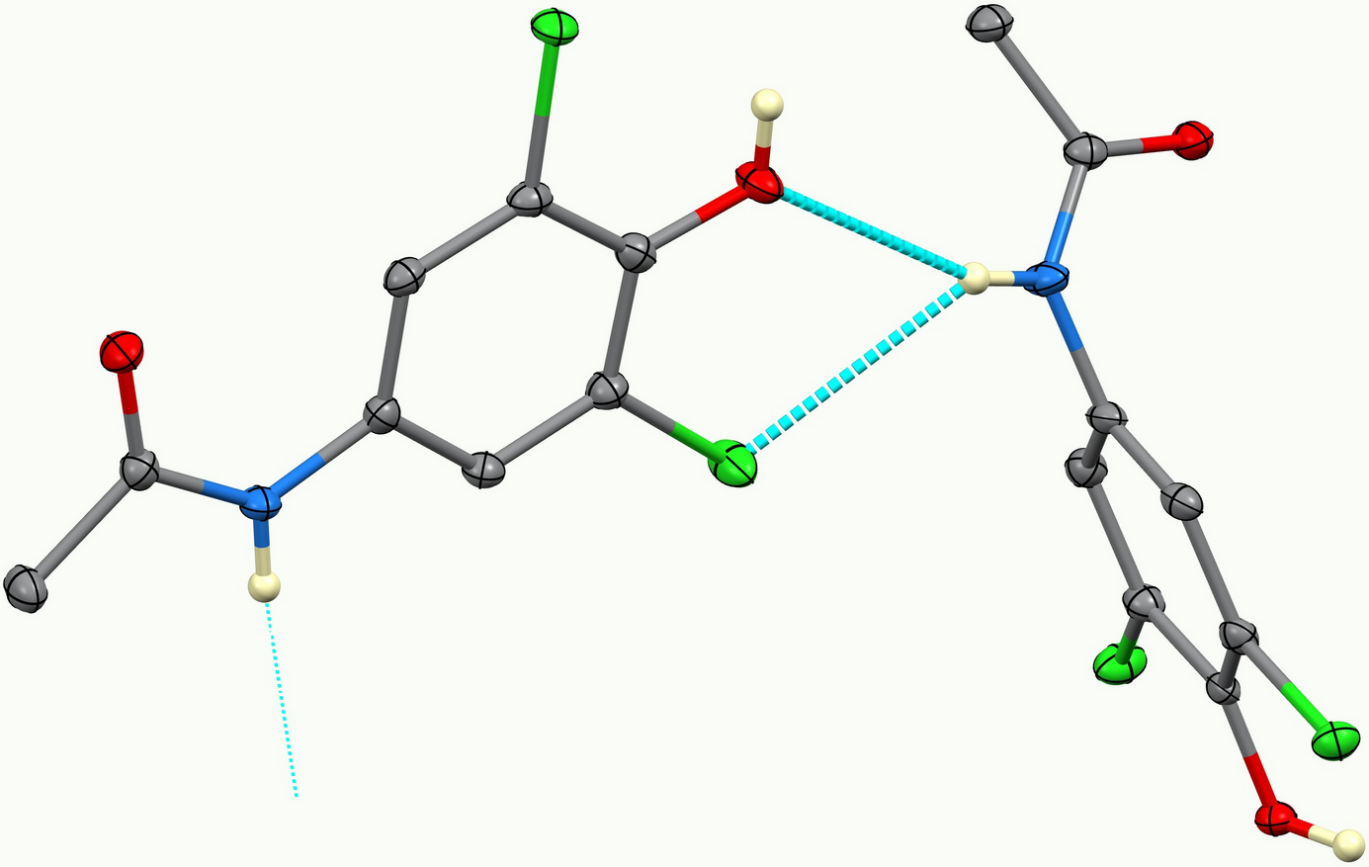
Detail of the bifurcated O—H⋯(O,Cl) hydrogen-bonding motif observed in the crystal packing of (**I**).

In the extended structure of (**I**), the mol­ecules are linked by numerous hydrogen bonds (Table 1 ▸). Among these, the two planar mol­ecules form anti­parallel hydrogen-bonded chains through N—H⋯O inter­actions of the acetamide substituents. The third, nonplanar mol­ecule links adjacent chains *via* additional bifurcated O—H⋯(O,Cl) inter­actions, generating a three-dimensional hydrogen-bonded network that consolidates the crystal packing (Fig. 2 ▸). Figs. 3 ▸ and 4 ▸ show, respectively, selected bifurcated hydrogen-bonding motifs and a view of the unit cell along the *a-*axis direction.

## Synthesis and crystallization

The title compound was synthesized by acetyl­ation of 4-amino-2,6-phenol (CAS 5930–28-9; purity: 97%) using acetic anhydride in acetic acid solvent: 1.78 g (10 mmol) of 4-amino-2,6-phenol in 10 ml of glacial acetic was allowed to react with 1.23 g (12 mmol) of acetic anhydride for 24–48 h at room temperature. The reaction mixture was stirred continuously during the reaction. In the end, the mixture was dried under vacuum, and the residue was purified by recrystallization once from aqueous solution. Single crystals of (**I**) in the form of colorless needles were grown in water by slow cooling of a hot and nearly saturated solution.

## Refinement

Crystal data, data collection and structure refinement details are summarized in Table 2 ▸.

## Supplementary Material

Crystal structure: contains datablock(s) I. DOI: 10.1107/S2414314626000751/hb4553sup1.cif

Structure factors: contains datablock(s) I. DOI: 10.1107/S2414314626000751/hb4553Isup2.hkl

Supporting information file. DOI: 10.1107/S2414314626000751/hb4553Isup3.cml

CCDC reference: 2525665

Additional supporting information:  crystallographic information; 3D view; checkCIF report

## Figures and Tables

**Figure 4:**
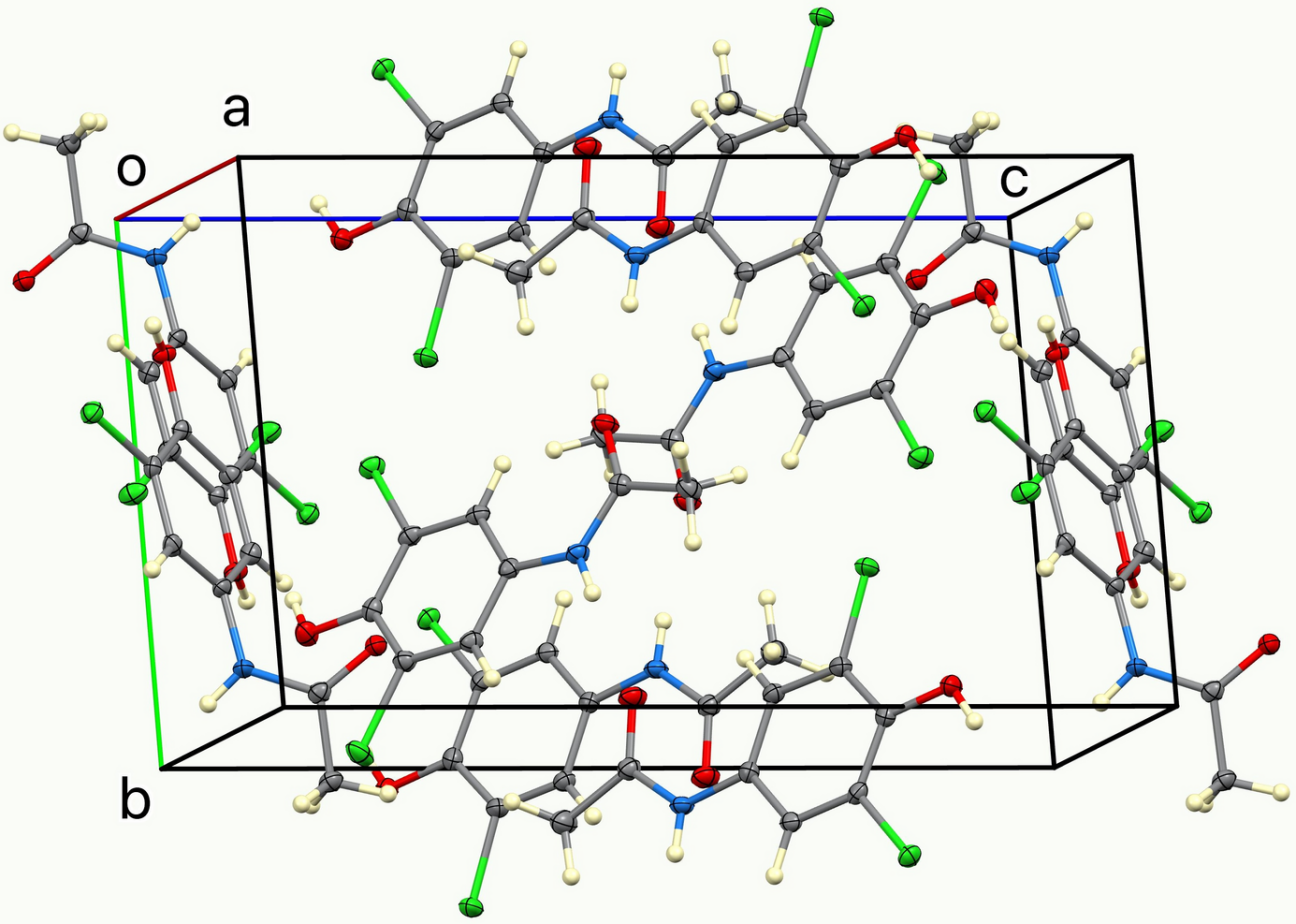
View of the unit cell of (**I**) along the *a*-axis direction.

**Table 1:** Hydrogen-bond geometry (Å, °)

*D*—H⋯*A*	*D*—H	H⋯*A*	*D*⋯*A*	*D*—H⋯*A*
O1—H10*H*⋯O2^i^	0.83 (2)	1.94 (2)	2.6776 (18)	148 (2)
N1—H1*N*⋯Cl3^ii^	0.84 (2)	2.78 (2)	3.3925 (17)	131 (2)
N1—H1*N*⋯O3^ii^	0.84 (2)	2.60 (2)	3.401 (2)	161 (2)
C8—H8*C*⋯O1^ii^	0.98	2.60	3.521 (2)	157
O3—H30*H*⋯O1	0.81 (2)	2.03 (2)	2.7602 (18)	149 (3)
O3—H30*H*⋯Cl4	0.81 (2)	2.59 (2)	3.0528 (14)	118 (2)
N2—H2*N*⋯O6^iii^	0.84 (2)	2.09 (2)	2.920 (2)	172 (2)
C13—H13⋯O4	0.95	2.26	2.866 (2)	121
O5—H50*H*⋯O2^i^	0.81 (2)	2.22 (2)	2.9546 (18)	150 (3)
O5—H50*H*⋯Cl5	0.81 (2)	2.51 (2)	2.9949 (14)	119 (2)
N3—H3*N*⋯O4^iv^	0.84 (2)	2.08 (2)	2.913 (2)	176 (2)
C21—H21⋯O6	0.95	2.24	2.853 (2)	121
C24—H24*A*⋯O4^iv^	0.98	2.59	3.464 (2)	149

Symmetry codes: (i) 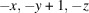; (ii) 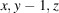; (iii) 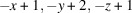; (iv) 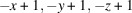.

**Table 2:** Experimental details

Crystal data	
Chemical formula	C_8_H_7_Cl_2_NO_2_
*M*_r_	220.05
Crystal system, space group	Triclinic, *P* 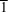
Temperature (K)	100
*a*, *b*, *c* (Å)	9.6844 (14), 9.8884 (16), 14.976 (3)
α, β, γ (°)	85.181 (10), 76.43 (1), 74.769 (7)
*V* (Å^3^)	1344.8 (4)
*Z*	6
Radiation type	Cu *K*α
μ (mm^−1^)	6.24
Crystal size (mm)	0.20 × 0.15 × 0.01

Data collection	
Diffractometer	Bruker D8 Venture DUO with Photon III C14
Absorption correction	Multi-scan (*SADABS*; Krause *et al.*, 2015 ▸)
*T*_min_, *T*_max_	0.625, 0.940
No. of measured, independent and observed [*I* > 2σ(*I*)] reflections	31306, 5786, 5206
*R*_int_	0.053
(sin θ/λ)_max_ (Å^−1^)	0.640

Refinement	
*R*[*F*^2^ > 2σ(*F*^2^)], *wR*(*F*^2^), *S*	0.032, 0.089, 1.06
No. of reflections	5786
No. of parameters	373
No. of restraints	6
H-atom treatment	H atoms treated by a mixture of independent and constrained refinement
Δρ_max_, Δρ_min_ (e Å^−3^)	0.55, −0.39

Computer programs: *APEX5* and *SAINT* (Bruker, 2016 ▸), *SHELXT2018/2* (Sheldrick, 2015*a* ▸), *SHELXL2019/1* (Sheldrick, 2015*b* ▸) and *Mercury* (Macrae *et al.*, 2020 ▸).

## full crystallographic data

### N-(3,5-Dichloro-4-hydroxyphenyl)acetamide Crystal data

**Table:**

C_8_H_7_Cl_2_NO_2_	*Z* = 6
*M**_r_* = 220.05	*F*(000) = 672
Triclinic, *P*1	*D*_x_ = 1.630 Mg m^−^^3^
*a* = 9.6844 (14) Å	Cu *K*α radiation, λ = 1.54184 Å
*b* = 9.8884 (16) Å	Cell parameters from 9884 reflections
*c* = 14.976 (3) Å	θ = 3.0–79.7°
α = 85.181 (10)°	µ = 6.24 mm^−^^1^
β = 76.43 (1)°	*T* = 100 K
γ = 74.769 (7)°	Plate, colourless
*V* = 1344.8 (4) Å^3^	0.20 × 0.15 × 0.01 mm

### N-(3,5-Dichloro-4-hydroxyphenyl)acetamide Data collection

**Table:**

Bruker D8 Venture DUO with Photon III C14 diffractometer	5206 reflections with *I* > 2σ(*I*)
Radiation source: IµS 3.0 microfocus	*R*_int_ = 0.053
φ and ω scans	θ_max_ = 80.5°, θ_min_ = 3.0°
Absorption correction: multi-scan (SADABS; Krause *et al.*, 2015)	*h* = −12→12
*T*_min_ = 0.625, *T*_max_ = 0.940	*k* = −12→12
31306 measured reflections	*l* = −18→19
5786 independent reflections	

### N-(3,5-Dichloro-4-hydroxyphenyl)acetamide Refinement

**Table:**

Refinement on *F*^2^	6 restraints
Least-squares matrix: full	Hydrogen site location: mixed
*R*[*F*^2^ > 2σ(*F*^2^)] = 0.032	H atoms treated by a mixture of independent and constrained refinement
*wR*(*F*^2^) = 0.089	*w* = 1/[σ^2^(*F*_o_^2^) + (0.0476*P*)^2^ + 0.6745*P*] where *P* = (*F*_o_^2^ + 2*F*_c_^2^)/3
*S* = 1.06	(Δ/σ)_max_ = 0.001
5786 reflections	Δρ_max_ = 0.55 e Å^−^^3^
373 parameters	Δρ_min_ = −0.39 e Å^−^^3^

### N-(3,5-Dichloro-4-hydroxyphenyl)acetamide Special details

**Table:**

Geometry. All esds (except the esd in the dihedral angle between two l.s. planes) are estimated using the full covariance matrix. The cell esds are taken into account individually in the estimation of esds in distances, angles and torsion angles; correlations between esds in cell parameters are only used when they are defined by crystal symmetry. An approximate (isotropic) treatment of cell esds is used for estimating esds involving l.s. planes.
Refinement. All non-H atoms were refined anisotropically. H atoms were treated by a mixed independent and riding model.

### N-(3,5-Dichloro-4-hydroxyphenyl)acetamide Fractional atomic coordinates and isotropic or equivalent isotropic displacement parameters (Å^2^)

**Table:**

	*x*	*y*	*z*	*U*_iso_*/*U*_eq_	
Cl1	0.35134 (4)	0.53881 (4)	−0.05637 (3)	0.01871 (10)	
Cl2	−0.13004 (4)	0.52183 (4)	0.20588 (3)	0.01605 (10)	
O1	0.09880 (13)	0.65297 (12)	0.08713 (8)	0.0140 (2)	
H10H	0.0124 (19)	0.696 (2)	0.1091 (16)	0.021*	
O2	0.13322 (14)	0.12695 (13)	−0.12814 (8)	0.0169 (3)	
N1	0.17559 (17)	0.08975 (15)	0.01623 (10)	0.0161 (3)	
H1N	0.204 (3)	0.029 (2)	0.0552 (15)	0.019*	
C1	0.11189 (18)	0.51671 (17)	0.07256 (11)	0.0124 (3)	
C2	0.23029 (18)	0.44736 (18)	0.00553 (12)	0.0141 (3)	
C3	0.25114 (19)	0.30765 (18)	−0.01319 (12)	0.0151 (3)	
H3	0.332077	0.262650	−0.059234	0.018*	
C4	0.15275 (19)	0.23420 (17)	0.03591 (12)	0.0143 (3)	
C5	0.03595 (19)	0.29868 (18)	0.10440 (11)	0.0142 (3)	
H5	−0.030199	0.247607	0.138824	0.017*	
C6	0.01692 (19)	0.43888 (18)	0.12201 (11)	0.0134 (3)	
C7	0.17477 (18)	0.04592 (18)	−0.06649 (12)	0.0143 (3)	
C8	0.2294 (2)	−0.10968 (18)	−0.07887 (12)	0.0171 (3)	
H8A	0.329501	−0.141133	−0.068846	0.026*	
H8B	0.229353	−0.131997	−0.141389	0.026*	
H8C	0.165136	−0.157433	−0.034474	0.026*	
Cl3	0.40021 (5)	1.01733 (5)	0.16640 (3)	0.02288 (11)	
Cl4	0.33207 (4)	0.48954 (4)	0.21640 (3)	0.01722 (10)	
O3	0.28764 (15)	0.78676 (14)	0.13405 (9)	0.0186 (3)	
H30H	0.254 (3)	0.722 (2)	0.1269 (18)	0.028*	
O4	0.65977 (15)	0.44791 (13)	0.43476 (9)	0.0194 (3)	
N2	0.63076 (16)	0.68011 (15)	0.39607 (10)	0.0150 (3)	
H2N	0.652 (3)	0.754 (2)	0.4057 (16)	0.018*	
C9	0.37032 (18)	0.75224 (18)	0.19782 (11)	0.0147 (3)	
C10	0.43264 (19)	0.85323 (18)	0.22059 (12)	0.0151 (3)	
C11	0.51803 (18)	0.82759 (18)	0.28510 (12)	0.0144 (3)	
H11	0.558929	0.898799	0.298694	0.017*	
C12	0.54439 (18)	0.69736 (18)	0.33037 (12)	0.0136 (3)	
C13	0.48653 (18)	0.59321 (18)	0.30858 (12)	0.0142 (3)	
H13	0.504517	0.503491	0.338150	0.017*	
C14	0.40183 (18)	0.62223 (18)	0.24281 (12)	0.0144 (3)	
C15	0.68395 (18)	0.56115 (18)	0.44264 (12)	0.0146 (3)	
C16	0.7786 (2)	0.57841 (19)	0.50564 (13)	0.0194 (4)	
H16A	0.781445	0.676874	0.504637	0.029*	
H16B	0.878336	0.519833	0.484830	0.029*	
H16C	0.737581	0.549727	0.568375	0.029*	
Cl5	−0.00475 (5)	0.72743 (4)	0.31367 (3)	0.01681 (10)	
Cl6	0.03404 (6)	1.25666 (4)	0.32909 (3)	0.02303 (11)	
O5	−0.05222 (15)	1.03136 (13)	0.25812 (9)	0.0190 (3)	
H50H	−0.069 (3)	0.965 (2)	0.2380 (18)	0.028*	
O6	0.32528 (15)	1.05309 (13)	0.56203 (10)	0.0208 (3)	
N3	0.28449 (16)	0.85184 (15)	0.52262 (10)	0.0152 (3)	
H3N	0.298 (3)	0.766 (2)	0.5329 (16)	0.018*	
C17	0.02825 (19)	0.98609 (18)	0.32290 (11)	0.0149 (3)	
C18	0.06138 (19)	0.84906 (18)	0.35663 (12)	0.0141 (3)	
C19	0.14531 (19)	0.80609 (18)	0.42180 (12)	0.0141 (3)	
H19	0.166334	0.710941	0.442481	0.017*	
C20	0.19856 (18)	0.90281 (18)	0.45676 (11)	0.0136 (3)	
C21	0.16516 (19)	1.04206 (18)	0.42647 (12)	0.0150 (3)	
H21	0.200021	1.109642	0.450257	0.018*	
C22	0.0804 (2)	1.08122 (18)	0.36118 (12)	0.0160 (3)	
C23	0.34095 (19)	0.92514 (19)	0.57035 (12)	0.0162 (3)	
C24	0.4274 (2)	0.8378 (2)	0.63615 (14)	0.0233 (4)	
H24A	0.427483	0.739182	0.632890	0.035*	
H24B	0.382235	0.870236	0.698858	0.035*	
H24C	0.528572	0.847223	0.619395	0.035*	

### N-(3,5-Dichloro-4-hydroxyphenyl)acetamide Atomic displacement parameters (Å^2^)

**Table:**

	*U*^11^	*U*^22^	*U*^33^	*U*^12^	*U*^13^	*U*^23^
Cl1	0.0173 (2)	0.0159 (2)	0.0224 (2)	−0.00726 (15)	0.00099 (15)	−0.00318 (15)
Cl2	0.01757 (19)	0.01529 (19)	0.01441 (19)	−0.00439 (15)	−0.00074 (15)	−0.00249 (14)
O1	0.0149 (6)	0.0111 (6)	0.0170 (6)	−0.0038 (5)	−0.0042 (5)	−0.0025 (4)
O2	0.0232 (6)	0.0122 (6)	0.0165 (6)	−0.0030 (5)	−0.0086 (5)	−0.0007 (5)
N1	0.0260 (8)	0.0093 (7)	0.0143 (7)	−0.0043 (6)	−0.0075 (6)	0.0002 (5)
C1	0.0144 (8)	0.0112 (8)	0.0143 (8)	−0.0035 (6)	−0.0077 (6)	−0.0007 (6)
C2	0.0154 (8)	0.0135 (8)	0.0152 (8)	−0.0060 (6)	−0.0047 (6)	0.0008 (6)
C3	0.0167 (8)	0.0148 (8)	0.0138 (8)	−0.0034 (6)	−0.0040 (6)	−0.0012 (6)
C4	0.0209 (8)	0.0103 (8)	0.0145 (8)	−0.0041 (6)	−0.0088 (7)	−0.0011 (6)
C5	0.0184 (8)	0.0144 (8)	0.0131 (8)	−0.0076 (6)	−0.0065 (6)	0.0011 (6)
C6	0.0162 (8)	0.0150 (8)	0.0102 (7)	−0.0040 (6)	−0.0049 (6)	−0.0009 (6)
C7	0.0146 (8)	0.0140 (8)	0.0152 (8)	−0.0056 (6)	−0.0028 (6)	−0.0012 (6)
C8	0.0216 (9)	0.0125 (8)	0.0190 (8)	−0.0052 (7)	−0.0062 (7)	−0.0019 (6)
Cl3	0.0330 (2)	0.0166 (2)	0.0271 (2)	−0.01274 (18)	−0.01799 (19)	0.00836 (16)
Cl4	0.0192 (2)	0.0145 (2)	0.0218 (2)	−0.00792 (15)	−0.00755 (16)	−0.00149 (15)
O3	0.0243 (7)	0.0177 (6)	0.0196 (6)	−0.0108 (5)	−0.0112 (5)	0.0022 (5)
O4	0.0244 (7)	0.0116 (6)	0.0251 (7)	−0.0050 (5)	−0.0113 (5)	0.0020 (5)
N2	0.0179 (7)	0.0115 (7)	0.0182 (7)	−0.0054 (6)	−0.0073 (6)	−0.0010 (6)
C9	0.0143 (8)	0.0170 (8)	0.0134 (8)	−0.0048 (6)	−0.0031 (6)	−0.0021 (6)
C10	0.0168 (8)	0.0130 (8)	0.0164 (8)	−0.0048 (6)	−0.0045 (6)	0.0003 (6)
C11	0.0151 (8)	0.0133 (8)	0.0163 (8)	−0.0052 (6)	−0.0042 (6)	−0.0012 (6)
C12	0.0126 (7)	0.0129 (8)	0.0147 (8)	−0.0020 (6)	−0.0028 (6)	−0.0020 (6)
C13	0.0141 (8)	0.0115 (8)	0.0164 (8)	−0.0037 (6)	−0.0015 (6)	−0.0013 (6)
C14	0.0143 (8)	0.0129 (8)	0.0170 (8)	−0.0056 (6)	−0.0016 (6)	−0.0029 (6)
C15	0.0145 (8)	0.0139 (8)	0.0152 (8)	−0.0034 (6)	−0.0031 (6)	−0.0001 (6)
C16	0.0224 (9)	0.0170 (9)	0.0221 (9)	−0.0062 (7)	−0.0116 (7)	0.0039 (7)
Cl5	0.0224 (2)	0.01470 (19)	0.01636 (19)	−0.00743 (15)	−0.00643 (15)	−0.00220 (14)
Cl6	0.0402 (3)	0.01053 (19)	0.0213 (2)	−0.00473 (17)	−0.01516 (19)	0.00195 (15)
O5	0.0283 (7)	0.0134 (6)	0.0187 (6)	−0.0043 (5)	−0.0130 (5)	−0.0001 (5)
O6	0.0262 (7)	0.0128 (6)	0.0278 (7)	−0.0062 (5)	−0.0127 (6)	−0.0017 (5)
N3	0.0190 (7)	0.0089 (7)	0.0190 (7)	−0.0031 (6)	−0.0074 (6)	−0.0001 (5)
C17	0.0173 (8)	0.0153 (8)	0.0119 (7)	−0.0031 (7)	−0.0034 (6)	−0.0014 (6)
C18	0.0161 (8)	0.0142 (8)	0.0127 (7)	−0.0055 (6)	−0.0011 (6)	−0.0039 (6)
C19	0.0154 (8)	0.0114 (8)	0.0146 (8)	−0.0029 (6)	−0.0019 (6)	−0.0007 (6)
C20	0.0157 (8)	0.0134 (8)	0.0118 (7)	−0.0036 (6)	−0.0029 (6)	−0.0009 (6)
C21	0.0193 (8)	0.0122 (8)	0.0142 (8)	−0.0052 (6)	−0.0033 (6)	−0.0016 (6)
C22	0.0219 (8)	0.0109 (8)	0.0147 (8)	−0.0035 (7)	−0.0038 (7)	−0.0005 (6)
C23	0.0174 (8)	0.0147 (8)	0.0175 (8)	−0.0040 (7)	−0.0052 (7)	−0.0018 (6)
C24	0.0298 (10)	0.0171 (9)	0.0274 (10)	−0.0048 (8)	−0.0158 (8)	−0.0007 (7)

### N-(3,5-Dichloro-4-hydroxyphenyl)acetamide Geometric parameters (Å, º)

**Table:**

Cl1—C2	1.7197 (17)	C10—C11	1.380 (2)
Cl2—C6	1.7311 (17)	C11—C12	1.395 (2)
O1—C1	1.351 (2)	C11—H11	0.9500
O1—H10H	0.833 (16)	C12—C13	1.388 (2)
O2—C7	1.240 (2)	C13—C14	1.391 (2)
N1—C7	1.349 (2)	C13—H13	0.9500
N1—C4	1.432 (2)	C15—C16	1.508 (2)
N1—H1N	0.838 (18)	C16—H16A	0.9800
C1—C6	1.394 (2)	C16—H16B	0.9800
C1—C2	1.398 (2)	C16—H16C	0.9800
C2—C3	1.386 (2)	Cl5—C18	1.7317 (17)
C3—C4	1.386 (2)	Cl6—C22	1.7303 (18)
C3—H3	0.9500	O5—C17	1.359 (2)
C4—C5	1.387 (2)	O5—H50H	0.812 (17)
C5—C6	1.389 (2)	O6—C23	1.233 (2)
C5—H5	0.9500	N3—C23	1.347 (2)
C7—C8	1.502 (2)	N3—C20	1.414 (2)
C8—H8A	0.9800	N3—H3N	0.835 (18)
C8—H8B	0.9800	C17—C18	1.388 (2)
C8—H8C	0.9800	C17—C22	1.395 (2)
Cl3—C10	1.7381 (18)	C18—C19	1.384 (2)
Cl4—C14	1.7357 (17)	C19—C20	1.387 (2)
O3—C9	1.353 (2)	C19—H19	0.9500
O3—H30H	0.814 (17)	C20—C21	1.391 (2)
O4—C15	1.223 (2)	C21—C22	1.387 (3)
N2—C15	1.359 (2)	C21—H21	0.9500
N2—C12	1.407 (2)	C23—C24	1.511 (3)
N2—H2N	0.836 (18)	C24—H24A	0.9800
C9—C14	1.395 (2)	C24—H24B	0.9800
C9—C10	1.396 (2)	C24—H24C	0.9800
			
C1—O1—H10H	112.4 (17)	C12—C13—C14	118.97 (16)
C7—N1—C4	123.14 (15)	C12—C13—H13	120.5
C7—N1—H1N	118.3 (17)	C14—C13—H13	120.5
C4—N1—H1N	117.9 (17)	C13—C14—C9	123.16 (16)
O1—C1—C6	124.18 (15)	C13—C14—Cl4	117.92 (13)
O1—C1—C2	118.36 (15)	C9—C14—Cl4	118.92 (14)
C6—C1—C2	117.44 (15)	O4—C15—N2	123.95 (16)
C3—C2—C1	121.68 (16)	O4—C15—C16	121.55 (16)
C3—C2—Cl1	119.26 (13)	N2—C15—C16	114.50 (15)
C1—C2—Cl1	119.05 (13)	C15—C16—H16A	109.5
C2—C3—C4	119.35 (16)	C15—C16—H16B	109.5
C2—C3—H3	120.3	H16A—C16—H16B	109.5
C4—C3—H3	120.3	C15—C16—H16C	109.5
C3—C4—C5	120.55 (15)	H16A—C16—H16C	109.5
C3—C4—N1	118.90 (16)	H16B—C16—H16C	109.5
C5—C4—N1	120.54 (16)	C17—O5—H50H	109.9 (19)
C4—C5—C6	119.17 (16)	C23—N3—C20	128.15 (15)
C4—C5—H5	120.4	C23—N3—H3N	118.5 (16)
C6—C5—H5	120.4	C20—N3—H3N	113.3 (16)
C5—C6—C1	121.77 (15)	O5—C17—C18	124.46 (16)
C5—C6—Cl2	119.79 (13)	O5—C17—C22	119.62 (15)
C1—C6—Cl2	118.43 (13)	C18—C17—C22	115.91 (16)
O2—C7—N1	123.09 (16)	C19—C18—C17	122.96 (16)
O2—C7—C8	122.14 (15)	C19—C18—Cl5	119.16 (13)
N1—C7—C8	114.77 (15)	C17—C18—Cl5	117.88 (13)
C7—C8—H8A	109.5	C18—C19—C20	119.59 (16)
C7—C8—H8B	109.5	C18—C19—H19	120.2
H8A—C8—H8B	109.5	C20—C19—H19	120.2
C7—C8—H8C	109.5	C19—C20—C21	119.39 (16)
H8A—C8—H8C	109.5	C19—C20—N3	116.74 (15)
H8B—C8—H8C	109.5	C21—C20—N3	123.87 (16)
C9—O3—H30H	110.7 (19)	C22—C21—C20	119.35 (16)
C15—N2—C12	128.27 (15)	C22—C21—H21	120.3
C15—N2—H2N	118.2 (16)	C20—C21—H21	120.3
C12—N2—H2N	113.5 (16)	C21—C22—C17	122.76 (16)
O3—C9—C14	125.72 (15)	C21—C22—Cl6	118.03 (13)
O3—C9—C10	118.25 (15)	C17—C22—Cl6	119.18 (14)
C14—C9—C10	116.03 (16)	O6—C23—N3	123.84 (17)
C11—C10—C9	122.28 (16)	O6—C23—C24	121.59 (16)
C11—C10—Cl3	118.96 (13)	N3—C23—C24	114.58 (16)
C9—C10—Cl3	118.76 (14)	C23—C24—H24A	109.5
C10—C11—C12	120.13 (16)	C23—C24—H24B	109.5
C10—C11—H11	119.9	H24A—C24—H24B	109.5
C12—C11—H11	119.9	C23—C24—H24C	109.5
C13—C12—C11	119.41 (16)	H24A—C24—H24C	109.5
C13—C12—N2	123.90 (16)	H24B—C24—H24C	109.5
C11—C12—N2	116.70 (15)		
			
O1—C1—C2—C3	179.89 (15)	C11—C12—C13—C14	−0.9 (2)
C6—C1—C2—C3	1.7 (3)	N2—C12—C13—C14	179.62 (16)
O1—C1—C2—Cl1	−1.1 (2)	C12—C13—C14—C9	−0.7 (3)
C6—C1—C2—Cl1	−179.35 (13)	C12—C13—C14—Cl4	179.54 (13)
C1—C2—C3—C4	−0.3 (3)	O3—C9—C14—C13	−179.34 (16)
Cl1—C2—C3—C4	−179.28 (13)	C10—C9—C14—C13	1.7 (2)
C2—C3—C4—C5	−1.2 (3)	O3—C9—C14—Cl4	0.4 (2)
C2—C3—C4—N1	−179.79 (16)	C10—C9—C14—Cl4	−178.52 (13)
C7—N1—C4—C3	−61.8 (2)	C12—N2—C15—O4	−1.8 (3)
C7—N1—C4—C5	119.65 (19)	C12—N2—C15—C16	177.56 (16)
C3—C4—C5—C6	1.3 (3)	O5—C17—C18—C19	179.13 (16)
N1—C4—C5—C6	179.84 (15)	C22—C17—C18—C19	−2.3 (3)
C4—C5—C6—C1	0.1 (3)	O5—C17—C18—Cl5	−0.2 (2)
C4—C5—C6—Cl2	178.90 (13)	C22—C17—C18—Cl5	178.36 (13)
O1—C1—C6—C5	−179.70 (15)	C17—C18—C19—C20	0.7 (3)
C2—C1—C6—C5	−1.6 (2)	Cl5—C18—C19—C20	−179.88 (13)
O1—C1—C6—Cl2	1.5 (2)	C18—C19—C20—C21	0.8 (2)
C2—C1—C6—Cl2	179.64 (13)	C18—C19—C20—N3	−179.53 (15)
C4—N1—C7—O2	−10.9 (3)	C23—N3—C20—C19	−175.73 (17)
C4—N1—C7—C8	168.63 (16)	C23—N3—C20—C21	3.9 (3)
O3—C9—C10—C11	179.73 (16)	C19—C20—C21—C22	−0.6 (3)
C14—C9—C10—C11	−1.3 (3)	N3—C20—C21—C22	179.69 (16)
O3—C9—C10—Cl3	0.2 (2)	C20—C21—C22—C17	−1.0 (3)
C14—C9—C10—Cl3	179.25 (13)	C20—C21—C22—Cl6	177.07 (13)
C9—C10—C11—C12	−0.2 (3)	O5—C17—C22—C21	−178.93 (16)
Cl3—C10—C11—C12	179.27 (13)	C18—C17—C22—C21	2.4 (3)
C10—C11—C12—C13	1.3 (3)	O5—C17—C22—Cl6	3.0 (2)
C10—C11—C12—N2	−179.14 (15)	C18—C17—C22—Cl6	−175.66 (13)
C15—N2—C12—C13	6.5 (3)	C20—N3—C23—O6	−0.6 (3)
C15—N2—C12—C11	−172.98 (16)	C20—N3—C23—C24	179.64 (17)

### N-(3,5-Dichloro-4-hydroxyphenyl)acetamide Hydrogen-bond geometry (Å, º)

**Table:**

*D*—H···*A*	*D*—H	H···*A*	*D*···*A*	*D*—H···*A*
O1—H10*H*···O2^i^	0.83 (2)	1.94 (2)	2.6776 (18)	148 (2)
N1—H1*N*···Cl3^ii^	0.84 (2)	2.78 (2)	3.3925 (17)	131 (2)
N1—H1*N*···O3^ii^	0.84 (2)	2.60 (2)	3.401 (2)	161 (2)
C8—H8*C*···O1^ii^	0.98	2.60	3.521 (2)	157
O3—H30*H*···O1	0.81 (2)	2.03 (2)	2.7602 (18)	149 (3)
O3—H30*H*···Cl4	0.81 (2)	2.59 (2)	3.0528 (14)	118 (2)
N2—H2*N*···O6^iii^	0.84 (2)	2.09 (2)	2.920 (2)	172 (2)
C13—H13···O4	0.95	2.26	2.866 (2)	121
O5—H50*H*···O2^i^	0.81 (2)	2.22 (2)	2.9546 (18)	150 (3)
O5—H50*H*···Cl5	0.81 (2)	2.51 (2)	2.9949 (14)	119 (2)
N3—H3*N*···O4^iv^	0.84 (2)	2.08 (2)	2.913 (2)	176 (2)
C21—H21···O6	0.95	2.24	2.853 (2)	121
C24—H24*A*···O4^iv^	0.98	2.59	3.464 (2)	149

Symmetry codes: (i) −*x*, −*y*+1, −*z*; (ii) *x*, *y*−1, *z*; (iii) −*x*+1, −*y*+2, −*z*+1; (iv) −*x*+1, −*y*+1, −*z*+1.
